# USP13 Downregulation Distinguishes Malignant from Adjacent Non-Neoplastic Prostate Tissue and Suggests Altered PTEN-Related Regulatory Pathways in a Korean Cohort

**DOI:** 10.3390/life16050712

**Published:** 2026-04-22

**Authors:** Jae Heon Kim, Miho Song, Kwang Woo Lee, Suyeon Park, Eunkyung Han, Ahrim Moon, Yun Seob Song

**Affiliations:** 1 Department of Urology, School of Medicine, Soonchunhyang University, Seoul 04401, Republic of Korea; piacekjh@hanmail.net (J.H.K.); miho@schmc.ac.kr (M.S.); 2 Department of Urology, School of Medicine, Soonchunhyang University, Bucheon 14584, Republic of Korea; urolkw@schmc.ac.kr; 3 Academic Research Office, Department of Biostatistics, Soonchunhyang University Seoul Hospital, Seoul 04401, Republic of Korea; suyeon1002@schmc.ac.kr; 4 Department of Applied Statistics, Chung-Ang University, Seoul 06911, Republic of Korea; 5 Department of Pathology, School of Medicine, Soonchunhyang University, Bucheon 14584, Republic of Korea

**Keywords:** deubiquitinases, ubiquitin-specific protease 13, prostate cancer

## Abstract

Ubiquitin-specific protease 13 (USP13) is a deubiquitinating enzyme that stabilizes phosphatase and tensin homolog deleted on chromosome 10 (PTEN), a well-established tumor suppressor involved in PI3K/AKT signaling. This study aimed to evaluate the relationship between USP13 immunohistochemical staining intensity and clinicopathological factors associated with prostate cancer progression. USP13 staining was scored as grade 0 (negative), 1 (weak), 2 (moderate), or 3 (strong) in 242 prostate cancer tissues and 22 adjacent non-neoplastic control tissues. Higher USP13 grades were exhibited by adjacent non-neoplastic tissues than prostate carcinoma. In comparison, lower USP13 grades were observed in 88.6% of the neoplastic regions (*p* < 0.001). No differences in PSA level, Gleason’s score, disease stage, involvement of either the seminal vesicle or lymph nodes, surgical margin positivity, biochemical or clinical recurrence rates, or overall survival statistics were found. Cox proportional hazards modeling showed no significant association between USP13 expression and biochemical recurrence-free survival or overall survival. Kaplan–Meier analysis demonstrated no statistically significant differences in survival outcomes according to USP13 expression, although a descriptive trend was observed. USP13 immunohistochemical staining distinguished malignant prostate tissue from adjacent non-neoplastic tissue in tissue microarrays. However, USP13 expression was not independently associated with pathological aggressiveness or survival outcomes in this cohort.

## 1. Introduction

Phosphatase and tensin homolog deleted on chromosome 10 (PTEN) is a major tumor suppressor that functions as a critical negative regulator of the phosphoinositide-3-kinase (PI3K)/AKT signaling pathway and plays an essential role in controlling cellular proliferation, survival, metabolism, and tumor development [1,2,3,4,5]. PTEN acts as a lipid phosphatase that converts phosphatidylinositol-3,4,5-trisphosphate into phosphatidylinositol-4,5-bisphosphate, thereby antagonizing PI3K-mediated signaling cascades [2,6]. Through this mechanism, PTEN serves as a central molecular brake on oncogenic signaling pathways that promote tumorigenesis. Mutations, deletions, or functional inactivation of PTEN are among the most frequent molecular alterations observed in human malignancies, including prostate cancer [7,8,9,10,11].

In addition to genetic alterations, PTEN activity is tightly regulated by multiple post-translational mechanisms that control its stability, localization, and enzymatic activity. These mechanisms include phosphorylation, acetylation, sumoylation, and ubiquitination [12,13,14]. In particular, the ubiquitin-proteasome system (UPS) plays a major role in regulating PTEN protein turnover and functional activity [12,13,14]. Deubiquitinating enzymes (DUBs) have emerged as critical regulators of protein stability and signaling pathways in cancer biology, modulating key substrates involved in tumorigenesis [15,16,17]. Several E3 ubiquitin ligases, including NEDD4-1, XIAP, WWP2, and CHIP, have been reported to mediate PTEN polyubiquitination and proteasomal degradation [18,19,20,21]. Conversely, deubiquitinating enzymes can reverse PTEN ubiquitination and thereby stabilize PTEN protein levels.

Among PTEN-regulating deubiquitinases, OTUD3 has been reported to stabilize PTEN and suppress tumorigenesis [22].

Among these enzymes, ubiquitin-specific protease 13 (USP13) has been identified as a key PTEN-stabilizing deubiquitinase. USP13 directly binds to PTEN and removes polyubiquitin chains, thereby preventing PTEN degradation and maintaining its tumor-suppressive function [23]. Experimental studies in breast cancer models have demonstrated that loss of USP13 leads to PTEN downregulation, increased AKT phosphorylation, enhanced cellular proliferation, and tumor growth [23]. Conversely, restoration of USP13 expression suppresses malignant progression in PTEN-positive cancer cells [23]. These findings suggest that USP13 may function as an upstream regulator of PTEN stability and may contribute to tumor suppression through modulation of PTEN-dependent signaling pathways.

Although PTEN dysregulation is a well-established event in prostate cancer biology, the clinical significance of USP13 expression in prostate cancer tissues remains poorly understood. In particular, it is unclear whether altered USP13 expression is associated with malignant transformation of prostate tissue or with clinicopathological features of prostate cancer progression.

Therefore, the aim of the present study was to evaluate USP13 expression in prostate cancer tissues and adjacent non-neoplastic prostate tissues using immunohistochemistry and to investigate whether USP13 expression is associated with clinicopathological characteristics and clinical outcomes in patients with prostate cancer.

## 2. Materials and Methods

### 2.1. Patients and Specimens

The specimens used in this study were obtained from the hospital between 2002 and 2021. A total of 243 prostate cancer tissue samples were initially collected, of which 242 were evaluable for clinicopathological analysis. 115 consecutive unselected tissue samples extracted at the time of radical prostatectomy (RP) were fixed in formalin and embedded in paraffin, and used to create a tissue microarray (TMA). Six samples from neighboring benign tissue to the tumor were also included. TMAs, obtained from anonymous sources and comprising 128 samples of human prostate carcinoma, gathered at RP, together with 16 samples of adjacent non-neoplastic tissue, were also procured from AccuMax (ISU ABXIS Co., Ltd., Seongnam, Republic of Korea). A total of 22 neighboring benign tissues to the tumor tissue samples were used, comprising 6 samples from RP at our hospital and 16 samples purchased from ISU ABXIS Co. For each TMA set, two specialist pathologists reviewed the slides stained with hematoxylin and eosin in order to confirm that they had been correctly labeled as either malignant or non-malignant tissue. The criteria published by the International Union Against Cancer and the World Health Organization/ International Society of Urological Pathology were used to determine the cancer stage and Gleason score for each individual. Additional clinical data collected included the patient’s age, the presence of seminal or lymph node invasion, and serum PSA titers. Genetic variables, including BRCA1/2 mutation status, were not available in this historical cohort. Patients were monitored for a median period of 67.43 months (range: 0.30–162.10 months) following RP; serum PSA was assayed at intervals. The regional scientific ethics committee gave approval for the study. (Seoul hospital: 2017-02-002, Bucheon hospital: 2017-03-004, Cheonan hospital: 2017-03-031-024, Gumi hospital: 2017-03-031-002).

### 2.2. Immunohistochemical Staining of Tissue Microarrays

The TMAs obtained from patients with prostatic carcinoma and the controls were evaluated, and consecutive slices, 3 µm in thickness, were sectioned. A specific rabbit anti-USP13 polyclonal antibody (sc514416, Santa Cruz Biotechnology, Dallas, TX, USA) was the primary antibody selected for the immunohistochemical analysis of USP13 expression. ImagePro software (Media Cybernetics, Rockville, MD, USA; version 6.0) was utilized in order to measure the density of antibody staining; the average staining intensity and the proportion of cells exhibiting positive staining were recorded.

Benign tissue samples from the prostate were assigned as positive controls. Two core samples from each patient were encompassed within each TMA. The paraffin sections from the TMA were dewaxed and rehydrated with xylene and alcohol, respectively, and then submerged in 0.01 M tri-sodium citrate solution in Coplin jars. These were warmed in a traditional pressure cooker for 3 min, and then washed for 5 min in cold running water, followed by tris-buffered saline, pH 7.4. Overnight incubation was performed with a 1:100 dilution of the primary anti-USP13 antibody. The sections of tissue underwent staining firstly, with biotinylated anti-rabbit immunoglobulins, and secondly, with peroxidase-labeled streptavidin (Dako, Carpinteria, CA, USA). The substrate used to obtain a readable signal was diaminobenzidine chromogen. Negative controls were generated using incubations in which the specific antibody was either excluded or preadsorbed. The expression of USP13 was deliberately only assessed on tissue core samples that were sufficiently preserved. The reporting pathologist was blinded to clinical endpoint data. USP13 staining intensity was scored from grade 0 to grade 3, representing negative, weak, moderate, and strong staining, respectively.

### 2.3. Survival Analysis

A rise in serum PSA ≥ 0.2 ng/mL on a minimum of 2 serial assays separated by 3 months or more was considered to represent biochemical recurrence. Any malignant skeletal deposits identified on a radionuclide bone scan, or enlarged lymph nodes or deposits within the viscera observed on abdominal, pelvic, or thoracic computed tomography scanning were deemed indicative of clinical recurrence. The period of risk was defined as the date of the patient’s RP until either recurrence was diagnosed or the date of the patient’s final serum PSA assay. The last day of follow-up or final PSA investigation was the point of censorship, where patients were lost to follow-up. Overall survival (OS) time was documented, i.e., the period from RP to death from any cause.

### 2.4. Statistical Analysis

The baseline covariates were analyzed initially, and any relationships between the USP13 cohort and the factors influencing prognosis were assessed. The categorized data were analyzed with the use of chi-square or Fisher’s exact tests, and given in a descriptive statistical format, i.e., *n* (%). Continuous data were evaluated utilizing Student’s *t*-test or the Mann–Whitney U test, and presented as mean ± standard deviation (SD) or median (1st, 3rd quartile). A Kaplan–Meier survival analysis was carried out, encompassing the biochemical or clinical recurrence data and the OS statistics. The Kaplan–Meier technique was used to plot the OS curves, and a log-rank test was utilized for their comparison. In order to establish any independent relationships between OS and the expression of USP13, Cox’s proportional hazard regression analysis was performed to establish the independent prognostic effect of the USP13 cohort, according to the age of the patient when diagnosed, histological grade, disease stage, PSA level, positivity of surgical margin, and any extension to the lymph nodes. For continuous variables, hazard ratios were interpreted as the relative change in risk associated with a one-unit increase in the variable. The Statistical Package for Social Sciences, version 26, and Rex, version 3.5.0 (Resift Inc., Seoul, Republic of Korea) were used for statistical analyses. A *p* value < 0.05 was deemed to represent statistical significance.

## 3. Results

### 3.1. Clinicopathological Characteristics of Patients with Prostatic Cancer and USP13 Expression

**Figure 1:**
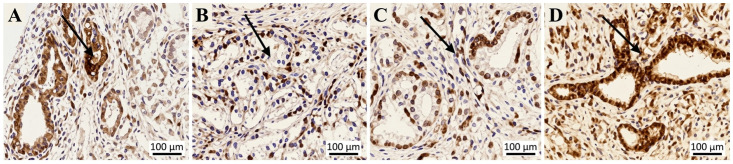
Representative immunohistochemical staining of USP13 in adjacent non-neoplastic and malignant prostate tissues. (**A**) Adjacent non-neoplastic prostate tissue showing strong USP13 expression (grade 3). (**B**) Prostate cancer tissue showing weak USP13 expression (grade 1). (**C**) Prostate cancer tissue showing moderate USP13 expression (grade 2). (**D**) Prostate cancer tissue showing strong USP13 expression (grade 3). Arrows indicate representative glandular epithelial cells along the luminal edge showing USP13 immunoreactivity. Scale bars = 100 μm.

A total of 265 TMAs were analyzed, including 243 prostate carcinoma tissues and 22 adjacent non-neoplastic tissues (Table 1). Of these, 264 TMAs were successfully evaluated for USP13 immunohistochemical staining. The baseline demographic characteristics, including age and body mass index (BMI), did not differ significantly between malignant and adjacent non-neoplastic tissue groups. USP13 staining scores of 3 and either 1 or 2 were exhibited by 46 and 218 samples, respectively. USP13 staining scores of 1 and either 2 or 3 were exhibited by 82 and 182 samples, respectively. In both group analyses, the higher grade was seen in the benign samples, and the lower grades in the samples from malignant tissue (*p* < 0.001) (Table 1, Figure 1).

USP13 expression grading, as either low (grades 1–2) or high (grade 3), was feasible in 242 of the patients with malignancy; one patient could not be analyzed owing to inadequate data (Table 2). The median patient age in this group was 65.0 years (60, 70). Serum PSA levels within this group were: ≤10, *n* = 168 (69.14%); 10–20, *n* = 40 (16.46%); >20, *n* = 35 (14.40%). The distribution of the Gleason score was as follows: ≤6, *n* = 24 (9.88%); 7, *n* = 107 (44.03%); 8–10, *n* = 112 (46.09%). Seminal invasion was present in 45 (18.60%). 241 (99.18%) patients had no lymph node involvement. Pathological stages of ≤T2 and ≥T3 were seen in 48 (19.75%) and 195 (80.25%) patients, respectively. No significant differences were observed between USP13 expression groups for most baseline clinicopathological variables (Table 2).

It was only possible to perform survival analysis in 115 subjects (Table 3) and for the presence of either biochemical or clinical recurrence and survival, in only 115. The median age of this group was 66.6 (64.5, 75.0) years. Serum PSA levels within this group were: ≤10, *n* = 53 (46.09%); 10–20, *n* = 35 (30.43%); >20, *n* = 27 (23.48%). The Gleason score was distributed as follows: ≤6, *n* = 20 (17.39%); 7, *n* = 64 (55.65%); 8–10, *n* = 31 (26.96%). A pathological stage of ≤T2 was present in 47 (40.87%) patients, and of ≥T3 in 68 (59.13%). Seminal invasion was evident in 14 (12.17%) patients and undetected in 101 (87.83%). 2 (1.74%) patients had lymph node involvement, 113 (98.26%) had no spread to the lymph nodes. Negative and positive surgical margins were recognized in 64 (55.65%) and 51 (44.35%), respectively. The different USP13 categories demonstrated no significant differences with respect to the baseline data. 14 (12.71%) of these patients presented with biochemical recurrence, of whom 12 were in grades 1–2, and 2 in grade 3. 3 patients were diagnosed with a clinical recurrence, all of which were in the USP13 grades 1–2 category. Fatality numbered 18 (20.48%), of whom 14 were in the USP13 grades 1–2 cohort, and 4 were in grade 3, showing no significant difference between the two groups. Even when the categories were reanalyzed by grouping grade 1 separately from grades 2 and 3, there was no significant difference between the two groups in terms of biochemical recurrence, clinical recurrence, or mortality.

### 3.2. Cox Proportional Hazard Model

#### 3.2.1. Biochemical Recurrence

The data from this analysis are presented in Table 4. Following univariate analysis, when compared with grade 3, the grade 1–2 cohort had a hazard ratio (HR) of 1.958 (95% CI: 0.239–16.029, *p* = 0.531), which failed to reach statistical significance. The hazard ratios for the PSA categories were as follows: 10–20, HR = 1.265 (95% CI 0.079–20.281, *p* = 0.868); >20, HR = 8.575 (95% CI: 0.130–71.340, *p* = 0.047). The statistical significance was not achieved even when the analysis was conducted by dividing the groups. Hazard ratios for pathological T stage and seminal vesicle invasion, which had a significant effect size, were 9.230 (95% CI: 1.135–75.084, *p* = 0.038) and 4.981 (95% CI: 1.233–20.121, *p* = 0.038).

Although a greater effect size was seen in relation to USP13 following multivariate analysis, it failed to reach significance (HR = 9.241, 95% CI: 0.973–87.815, *p* = 0.053). For continuous variables, each one-unit increase in height or weight corresponded to the estimated change in hazard shown in the model. The ultimate model additionally incorporated data for PSA level and pathological T stage: PSA 10–20, HR = 1.980 (95% CI: 0.103–38.059, *p* = 0.651); PSA > 20, HR = 11.852 (95% CI: 1.044–134.589, *p* = 0.046); pathological T stage, HR = 5.427 (95% CI: 0.577–51.081, *p* = 0.139).

#### 3.2.2. Overall Survival

The overall survival data are shown in Table 5. Following a comparison of the USP13 staining intensities, grade 1–2 versus grade 3, both univariate (HR = 1.666, 95% CI: 0.476–5.831, *p* = 0.425) and multivariate (HR = 1.938; 95% CI: 0.544–6.896, *p* = 0.307) analyses failed to demonstrate statistical significance, although a trend towards a rise was seen in the latter. With each year in age difference, the likelihood of death rose by a factor of 1.089 (95% CI: 0.987–1.202, *p* = 0.088).

#### 3.2.3. Kaplan–Meier Survival Analysis

**Figure 2:**
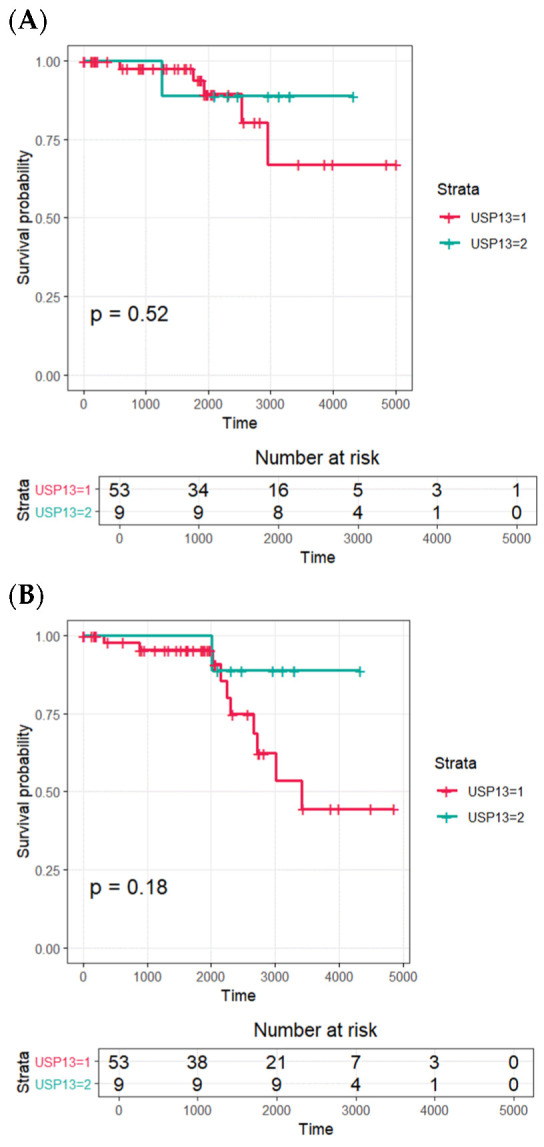
Kaplan–Meier survival analysis for USP13 expression in prostate cancer patients. (**A**) Biochemical recurrence-free survival. (**B**) Overall survival. Patients were stratified into low USP13 expression (grades 1–2) and high USP13 expression (grade 3). No statistically significant differences were observed between groups.

The Kaplan–Meier survival data and the results of the log-rank test for the expression of USP13 in patients with prostatic carcinoma are illustrated in Figure 2. Mean ± SD times to biochemical recurrence for the USP13 grade 3 and grades 1–2 groups were 11.10 ± 0.73 years and 11.94 ± 0.65 years (*p* = 0.351), respectively. Mean ± SD times to clinical recurrence for the USP13 grade 3 and grades 1–2 cohorts were 12.51 ± 0.47 years and 10.05 ± 0.68 years (*p* = 0.420), respectively. Kaplan–Meier analysis showed no statistically significant differences in biochemical recurrence-free survival or overall survival according to USP13 expression category, although lower USP13 expression showed a descriptive trend toward less favorable outcomes.

## 4. Discussion

In the samples examined in this research, USP13 staining grades 2 and 3 were seen in all the histology specimens of benign tissue obtained from areas adjacent to prostatic tumor tissue. In contrast, USP13 grades 1 and 2 were seen in 88.6% (*p* < 0.001) of samples containing malignant prostatic tissue (Table 1, Figure 1). This difference indicates that USP13 expression may help distinguish malignant from adjacent non-neoplastic prostate tissue.

Compared to individuals with a higher USP13 grade, patients exhibiting a lower USP13 grade failed to demonstrate more elevated serum PSA titers, a higher Gleason’s score, a more advanced disease stage, or additional lymph node or seminal vesicle involvement. Similarly, the survival analysis revealed that in comparison to their high-grade USP13 counterparts, the group with a low USP13 staining intensity did not exhibit increased serum PSA titers, a higher Gleason’s score, more advanced disease stage, lymph node or seminal vesicle involvement, a positive surgical margin, more frequent biochemical or clinical recurrence, or higher mortality rates. Given the immunohistochemical staining intensity for USP13, the prostate carcinoma prognosis was not anticipated (Table 2 and Table 3). Accordingly, USP13 expression was not a robust prognostic indicator in this cohort.

No correlations between USP13 expression, as determined using the immunohistochemical staining technique, and either freedom from biochemical recurrence or OS were found using Cox proportional hazard modeling (Table 4 and Table 5). Although Kaplan–Meier analysis suggested a descriptive trend toward poorer outcomes in the lower USP13 group, these differences did not reach statistical significance on log-rank testing (Figure 2). In this study, there was a low death rate amongst the studied cohort over the study’s follow-up period, which was assumed to be related to the small population of patients investigated.

Although in human neoplasms, the PTEN protein is commonly found to exhibit genetic variations, only a quarter of patients with malignancy demonstrate an association between PTEN protein absence and its mRNA loss [4,12,24], a finding which highlights the post-transcriptional and post-translational import of PTEN modulation. The expression, activity, and site of PTEN can be influenced by mono- or poly-ubiquitination, phosphorylation, sumoylation, and acetylation, and controlled by non-coding RNAs [4,12,13]. Contemporary research has demonstrated that both ubiquitination and deubiquitination processes moderate the characteristics and activity of PTEN [4,12,13].

USP13 catalyzes PTEN poly-ubiquitination reversal, stabilizing the protein and facilitating the suppression of malignancy [23,25]. Carcinogenesis is enhanced in the absence of USP13, occurring via PTEN downregulation. Consequently, the anti-cancer properties of USP13 vary according to the status of the PTEN protein. In human breast carcinoma, downregulation of USP13 has been observed, and correlates with PTEN protein concentrations [23]. In the present study, reduced USP13 expression in malignant prostate tissue is biologically consistent with altered PTEN-related regulatory pathways. However, PTEN immunohistochemistry, PTEN genomic status, and downstream signaling markers such as phosphorylated AKT were not directly assessed in our cohort. Therefore, the mechanistic link between USP13 downregulation and PTEN pathway dysregulation should be regarded as indirect rather than directly demonstrated.

This research demonstrated that tissue expression of USP13, using an immunohistochemical technique, can be used to distinguish benign and malignant tissues in samples obtained from patients with prostatic carcinoma. USP13 is mostly found within the cell cytoplasm or attached to the cell membrane [23], loci which are in keeping with its PTEN poly-ubiquitination reversal functions at these sites [23].

In comparison to p53, PTEN is a more stable protein. However, amplification of PTEN ubiquitin ligases, or PTEN deubiquitinase downregulation, can hasten its breakdown. In human breast carcinoma, downregulation of USP13 is evident, which is consistent with the degree of PTEN expression. Consequently, carcinogenesis may be promoted by the absence of USP13 in breast tissues exhibiting heterozygous PTEN inactivation [23]. Based on the known role of USP13 in PTEN stabilization, reduced USP13 expression in malignant tissue is biologically plausible in the context of prostate carcinogenesis. However, in our cohort, USP13 expression was not consistently associated with clinicopathological aggressiveness or survival outcomes.

However, contrary to our hypothesis, the correlation between USP13 expression and both tumor malignancy and clinical prognosis was not consistently observed, which may reflect the complex and heterogeneous genomic landscape of prostate cancer [26]. One possible explanation for USP13 not showing an association with clinical outcomes and pathological features is that this protein may play a role only in specific biological pathways, which may not be directly related to clinical prognosis. Although USP13 may be involved in particular metabolic processes or molecular variations within cancer cells, its effects might not align with the clinical indicators we investigated. Additionally, due to the complex biological nature of cancer, various factors can influence prognosis, and the role of USP13 could have been diluted by other variables. Another possible explanation is that while USP13 expression may generally be lower in malignant tissues compared to benign ones, its overexpression in specific cancer subgroups (e.g., those with high PSA levels or high Gleason scores) could indicate a role in cancer progression or aggressiveness. This dual behavior of USP13—low expression in most cancer tissues but higher expression in more aggressive cancers—presents an important area for further investigation.

In this study, we investigated the expression of USP13 in prostate cancer patients. Our study has several limitations. One limitation of our study is the relatively small number of patients included in the study, along with a relatively short follow-up period. Out of the total 265 tissue microarray TMA samples used in the study, only 115 patients were included in the survival analysis, which is relatively small. This may hinder the ability to sufficiently detect effect sizes in statistical analyses. This limitation could particularly explain the lack of statistical significance in the correlation between USP13 expression and survival or recurrence rates. Third, because this was a retrospective study, variables such as BMI, smoking history, and alcohol consumption history were not uniformly available across all cases. Fourth, genetic variables such as BRCA1/2 mutation status were unavailable in this historical cohort. Finally, the absence of direct PTEN-related analyses limits mechanistic interpretation of the USP13–PTEN axis.

Despite these limitations, our findings suggest that the role of USP13 in prostate cancer may be more complex than initially expected. USP13 appears to have value as a tissue-level discriminator between malignant and adjacent non-neoplastic prostate tissue, but its prognostic significance remains uncertain. Further studies integrating USP13 with PTEN immunohistochemistry, PTEN genomic assays, and downstream pathway analyses in larger, well-annotated cohorts are warranted.

## 5. Conclusions

In this study, adjacent non-neoplastic prostate tissue could be distinguished from malignant prostate tissue by USP13 staining intensity. USP13 immunohistochemical staining may therefore have adjunctive value in distinguishing malignant from adjacent non-neoplastic tissue components in prostate specimens. However, USP13 expression was not significantly associated with pathological aggressiveness or survival outcomes in this cohort.

## Figures and Tables

**Table 1:** Clinicopathological properties of the patient and USP13 expression in prostate cancer.

	Total (*n* = 264)	Prostate Cancer (*n* = 242)	Adjacent Non-Neoplastic Tissue (*n* = 22)	*p*-Value
Age (years)	65 (60, 70)	65 (60, 70)	66 (61, 71)	0.648 *
BMI (kg/m^2^)	24.6 ± 3.2	24.7 ± 3.3	24.3 ± 2.9	0.582 ##
USP13				<0.001
1	82 (31.06%)	82 (33.88%)	0 (0%)	
2	136 (51.52%)	132 (54.55%)	4 (18.18%)	
3	46 (17.42%)	28 (11.57%)	18 (81.82%)	
USP13_grade				<0.001
1–2	218 (82.58%)	214 (88.43%)	4 (18.18%)	
3	46 (17.42%)	28 (11.57%)	18 (81.82%)	
USP13_grade				<0.001
1	82 (31.06%)	82 (33.88%)	0 (0%)	
2–3	182 (68.94%)	160 (66.12%)	22 (100%)	

USP13 staining intensity was scored as follows: **grade 0, negative; grade 1, weak; grade 2, moderate; and grade 3, strong**. Age is presented as **median (interquartile range)** and BMI as **mean ± standard deviation**. Data are shown as ***n* (%) unless otherwise indicated**. The number of evaluable prostate tissues for USP13 immunohistochemistry was **264, including 242 prostate cancer tissues and 22 adjacent non-neoplastic tissues**. * Mann–Whitney U test, ## Student’s *t*-test.

**Table 2:** USP13 immunohistochemical staining in prostate cancer tissues from all evaluable patients.

	Total (*n* = 242)	USP13		*p*-Value	USP13		*p*-Value
		1–2 (*n* = 214)	3 (*n* = 28)		1 (*n* = 82)	2–3 (*n* = 160)	
Age (years)	65 (60, 70)	65 (60, 70)	66 (60, 72)	0.392 *	66 (60, 72)	66 (62, 72)	0.982 *
PSA (ng/mL)				0.016 **			0.327 **
≤10	168 (69.14%)	154 (71.96%)	13 (46.43%)		60 (73.17%)	107 (66.88%)	
10–20	40 (16.46%)	33 (15.42%)	7 (25.00%)		14 (19.44%)	26 (16.25%)	
>20	35 (14.4%)	27 (12.62%)	8 (28.57%)		8 (9.76%)	27 (16.88%)	
Gleason score				0.136 **			0.866 **
≤6	24 (9.88%)	22 (10.28%)	1 (3.57%)		8 (9.76%)	15 (9.38%)	
7	107 (44.03%)	98 (45.79%)	9 (32.14%)		38 (46.34%)	69 (43.13%)	
8–10	112 (46.09%)	94 (43.93%)	18 (64.29%)		36 (46.90%)	76 (47.50%)	
Pathological T stage				>0.99 ^#^			0.391 ^#^
≤T2	48 (19.75%)	42 (19.63%)	5 (17.86%)		13 (15.85%)	34 (21.25%)	
≥T3	172 (80.37%)	172 (80.37%)	23 (82.14%)		69 (84.15%)	126 (78.75%)	
Seminal vesicle invasion				0.373 ^#^			0.729 ^#^
Negative	197 (81.4%)	171 (80.28%)	25 (89.29%)		68 (82.93%)	128 (80.50%)	
Positive	45 (18.6%)	42 (19.72%)	3 (10.71%)		14 (17.07%)	31 (19.50%)	
Lymph node involvement				0.218 **			>0.99 **
Negative	241 (99.18%)	213 (99.53%)	27 (96.43%)		81 (98.78%)	159 (99.38%)	
Positive	2 (0.47%)	1 (0.47%)	1 (3.57%)		1 (1.22%)	1 (0.62%)	

USP13 staining intensity was scored as follows: grade 0, negative; grade 1, weak; grade 2, moderate; and grade 3, strong. Age is presented as median (interquartile range). Data are shown as *n* (%) unless otherwise indicated. The 242 patients included 114 institutional cases and 128 cases from purchased TMAs. BMI, smoking history, and alcohol consumption history were reviewed, where available. * Mann–Whitney U test; ** Fisher’s exact test; ^#^ chi-square test.

**Table 3:** USP13 immunohistochemical staining of prostate cancer and adjacent non-neoplastic tissues for radical prostatectomy due to prostate cancer in 115 patients with survival analysis.

	Total (*n* = 115)	USP13		*p*-Value	USP13		*p*-Value
		1–2 (*n* = 98)	3 (*n* = 17)		1 (*n* = 41)	2–3 (*n* = 74)	
Age (years)	69 (65, 75)	69 (64, 76)	69 (68, 75)	0.695 ^##^	71 (66, 75)	69 (64, 75)	0.637 ^##^
Height (cm)	165.53 ± 5.77	165.74 ± 5.92	164.3 ± 4.78	0.28 ^##^	165.61 ± 6.03	165.48 ± 5.66	0.910 ^##^
Weight (kg)	67.1 (60.9, 74.1)	68.2 (61.2, 75)	65.5 (55.8, 69.0)	0.226 *	68.79 ± 11.13	67.67 ± 9.88	0.593 *
BMI (kg/m^2^)	24.5 ± 3.1	24.8 ± 3.2	24.2 ± 2.9	0.311 *	25.1 ± 3.8	24.7 ± 3.4	0.602
PSA (ng/mL)				0.026 **			0.250 **
≤10	53 (46.09%)	50 (51.02%)	3 (17.65%)		23	30	
10–20	35 (30.43%)	28 (28.57%)	7 (41.18%)		11	24	
>20	27 (23.48%)	20 (20.41%)	7 (41.18%)		7	20	
Gleason score				0.119 **			0.206 **
≤6	20 (17.39%)	19 (19.39%)	1 (5.88%)		8	12	
7	64 (55.65%)	56 (57.14%)	8 (47.06%)		26	38	
8–10	31 (26.96%)	23 (23.47%)	8 (47.06%)		7	24	
Pathological T stage				0.439 ^#^			0.167 ^#^
≤T2	47 (40.87%)	42 (42.86%)	5 (29.41%)		13	34	
≥T3	68 (59.13%)	56 (57.14%)	12 (70.59%)		28	40	
Seminal vesicle invasion				>0.99 **			0.563 **
Negative	101 (87.83%)	86 (57.76%)	15 (88.24%)		35	66	
Positive	14 (18.6%)	12 (12.24%)	2 (11.76%)		6	8	
Lymph node involvement				0.275 **			>0.999 **
Negative	113 (98.26%)	97 (98.98%)	16 (94.12%)		40 (97.56%)	73 (98.65%)	
Positive	2 (1.74%)	1 (1.02%)	1 (5.88%)		1 (2.44%)	1 (1.35%)	
Surgical margin				0.984 ^#^			0.171 ^#^
Negative	64 (55.65%)	54 (55.10%)	10 (58.82%)		19 (46.34%)	45 (60.81%)	
Positive	51 (44.35%)	44 (44.90%)	7 (41.18%)		22 (53.66%)	29 (39.19%)	
Biochemical recurrence				>0.99 **			0.563 **
Negative	101 (87.83%)	86 (87.76%)	15 (88.24%)		35 (85.37%)	66 (89.19%)	
Positive	14 (12.17%)	12 (12.24%)	2 (11.76%)		6 (14.63%)	8 (10.81%)	
Clinical recurrence				>0.99 **			0.289 **
Negative	112 (97.39%)	95 (96.94%)	17 (100%)		39 (95.12%)	73 (98.65%)	
Positive	3 (2.61%)	3 (3.06%)	0 (0%)		2 (4.88%)	1 (1.35%)	
Mortality				0.303 **			0.594 **
No	97 (84.35%)	84 (85.71%)	13 (76.47%)		36 (87.80%)	61 (82.43%)	
Yes	18 (15.65%)	14 (14.29%)	4 (23.53%)		5 (12.20%)	13 (17.57%)	

USP13 staining intensity was scored as follows: grade 0, negative; grade 1, weak; grade 2, moderate; and grade 3, strong. Survival analysis was performed in 115 patients from the institutional radical prostatectomy cohort with available follow-up data. Continuous variables are presented as mean ± standard deviation or median (interquartile range), as appropriate. * Mann–Whitney U test; ** Fisher’s exact test; ^#^ chi-square test; ^##^ Student’s *t*-test.

**Table 4:** Cox proportional hazard modeling of USP13 after accounting for Biochemical recurrence free survival.

	Univariable Analysis				Multivariable Analysis							
					Model 1				Model 2			
	HR	95% CI		*p*-Value	HR	95% CI		*p*-Value	HR	95% CI		*p*-Value
		Lower	Upper			Lower	Upper			Lower	Upper	
Age (years)	1.052	0.974	1.136	0.197								
Height (cm)	0.929	0.844	1.023	0.134					0.872	0.777	0.979	0.020
Weight (kg)	1.028	0.984	1.074	0.223					1.061	1.009	1.117	0.021
USP13												
1	1.970	0.396	9.805	0.408	1.744	0.336	9.038	0.508				
2	1.720	0.341	8.680	0.512	2.110	0.400	11.133	0.379				
3	1.000				1.000							
USP13 grade												
1–2	1.841	0.408	8.311	0.428								
3	1.000								3.724	0.779	17.795	0.100
PSA (ng/mL)												
≤10	1.000								1.000			
10–20	1.728	0.384	7.778	0.476					1.040	0.210	5.144	0.962
>20	3.635	0.938	14.082	0.062					3.887	0.919	16.437	0.065
Gleason score												
≤6												
7												
8–10												
Pathological T stage												
≤T2	1.000				1.000				1.000			
≥T3	11.465	1.495	87.924	0.019	10.521	1.335	82.902	0.026	9.182	1.172	71.933	0.035
Seminal vesicle invasion												
Negative	1.000				1.000							
Positive	2.610	0.807	8.445	0.109	3.624	0.960	13.683	0.058				
Lymph node involvement												
Negative	1.000											
Positive	2.984	0.384	23.191	0.298								
Surgical margin												
Negative	1.000											
Positive	5.349	1.480	19.330	0.011								

USP13 staining intensity was scored as follows: grade 0, negative; grade 1, weak; grade 2, moderate; and grade 3, strong. Hazard ratios for continuous variables represent the relative change in hazard associated with a one-unit increase in the variable. Survival analysis was performed in 115 patients from the institutional radical prostatectomy cohort with available follow-up data.

**Table 5:** Cox proportional hazards modeling of USP13 for overall survival.

	Univariable Analysis				Multivariable Analysis							
					Model 1				Model 2			
	HR	95% CI		*p*-Value	HR	95% CI		*p*-Value	HR	95% CI		*p*-Value
		Lower	Upper			Lower	Upper			Lower	Upper	
Age (years)	1.064	0.981	1.154	0.135								
Height (cm)	0.955	0.885	1.032	0.246								
Weight (kg)	0.955	0.906	1.005	0.078	0.946	0.896	0.999	0.047	0.952	0.904	1.004	0.068
USP13												
1	1.120	0.297	4.218	0.868	1.080	0.287	4.070	0.910				
2	1.286	0.392	4.217	0.679	1.765	0.515	6.052	0.366				
3	1.000				1.000							
USP13 grade												
1–2	1.463	0.371	5.771	0.587					1.411	0.456	4.367	0.550
3	1.000								1.000			
PSA (ng/mL)												
≤10	1.000											
10–20	1.402	0.438	4.491	0.570								
>20	1.367	0.413	4.523	0.608								
Gleason score												
≤6	1.000											
7	0.726	0.218	2.422	0.602								
8–10	0.753	0.210	2.700	0.663								
Pathological T stage												
≤T2	1.000											
≥T3	1.347	0.521	3.484	0.539								
Seminal vesicle invasion												
Negative	1.000											
Positive	1.769	0.68	5.060	0.288								
Lymph node involvement												
Negative	1.000											
Positive	1.884	0.246	14.422	0.542								
Surgical margin												
Negative	1.000											
Positive	1.504	0.577	3.919	0.404								

USP13 staining intensity was scored as follows: grade 0, negative; grade 1, weak; grade 2, moderate; and grade 3, strong. Hazard ratios for continuous variables represent the relative change in hazard associated with a one-unit increase in the variable. Survival analysis was performed in 115 patients from the institutional radical prostatectomy cohort with available follow-up data.

## Data Availability

The data are not publicly available due to ethical restrictions. The data are, however, available from the corresponding author upon reasonable request.

## Abbreviations

The following abbreviations are used in this manuscript:

AKT	Protein kinase B
BMI	Body mass index
BRI	Body roundness index
HR	Hazard ratio
IHC	Immunohistochemistry
OS	Overall survival
PI3K	Phosphoinositide 3-kinase
PSA	Prostate-specific antigen
PTEN	Phosphatase and tensin homolog deleted on chromosome 10
TMA	Tissue microarray
UPS	Ubiquitin-proteasome system
USP13	Ubiquitin-specific protease 13
